# Innovative Health Technologies to Improve Emergency Department Performance: Dario Tedesco

**DOI:** 10.1093/eurpub/ckac131.169

**Published:** 2022-10-25

**Authors:** D Tedesco, A Capodici, G Gribaudo, Z Di Valerio, M Montalti, A Salussolia, V Barbagallo, M Rolli, MP Fantini, D Gori

**Affiliations:** 1 General Directorate of Healthcare and Welfare, Emilia-Romagna Region, Bologna, Italy; 2 Unit of Hygiene, University of Bologna, Bologna, Italy

## Abstract

**Background:**

Emergency Departments (EDs) are increasingly pivotal, with a constant increment in their use, despite stable or declining fundings. Crowding can lead to disruptions and the COVID-19 epidemic has further burdened ED services. However, the pandemic has seen an increased use of telemedicine and digital health tools, which may be notably beneficial for EDs. This study offers a review of the latest available digital health technologies and their effectiveness to improve ED performance.

**Methods:**

We performed a narrative review to identify digital and technological innovations in EDs. The themes of interest were defined in 4 areas: Patient Assessment, Patient Experience, Resource Allocation, and Discharge. Data was analyzed by 5 independent reviewers who focused on different macro-areas. Disagreement on data was discussed with 2 independent tiebreakers.

**Results:**

Our search yielded 25 articles addressing 4 topics: Patient Assessment, Resource Allocation, Patient Experience, Discharge. We found that digital tools and Artificial Intelligence are powerful tools to detect, collect, and process data from patients, to improve healthcare delivery in EDs. The Resource Allocation category showed to be key in optimizing services already in place. New technologies showed effective to improve Patient Experience by curbing pain and anxiety. Innovative technologies demonstrated efficacy after Discharge when patients need guidance from clinicians for follow-up care.

**Conclusions:**

Our review shows evidence of increasing effectiveness of innovative tools in reducing wait time and improving performance and patient experience in EDs. Technology applied to resource allocation appeared to be the most effective category. Prediction algorithms could be used to improve workforce allocation and bed management. Critical care systems must meet the challenge of innovative technologies which can lead to a new era in healthcare delivery with improvements for patients and healthcare professionals.

**Key messages:**

• Digital innovation will have a significant impact on several dimensions of healthcare in the near future.

• Healthcare systems and EDs must meet the challenge of innovative technologies which can lead to a new era in healthcare delivery with improvements for both patients and healthcare professionals.

